# Leveraging the Use of Historical Data Gathered During Seed Regeneration of an *ex Situ* Genebank Collection of Wheat

**DOI:** 10.3389/fpls.2018.00609

**Published:** 2018-05-08

**Authors:** Norman Philipp, Stephan Weise, Markus Oppermann, Andreas Börner, Andreas Graner, Jens Keilwagen, Benjamin Kilian, Yusheng Zhao, Jochen C. Reif, Albert W. Schulthess

**Affiliations:** ^1^Department of Breeding Research, Leibniz Institute of Plant Genetics and Crop Plant Research (IPK), Gatersleben, Germany; ^2^Department of Genebank, Leibniz Institute of Plant Genetics and Crop Plant Research (IPK), Gatersleben, Germany; ^3^Institute for Biosafety in Plant Biotechnology, Julius Kühn-Institut (JKI) – Federal Research Centre for Cultivated Plants, Quedlinburg, Germany; ^4^Global Crop Diversity Trust, Bonn, Germany

**Keywords:** genetic resources, genebank, winter wheat, historical data, bio-digital resource center, data quality assessment

## Abstract

Genebanks are a rich source of genetic variation. Most of this variation is absent in breeding programs but may be useful for further crop plant improvement. However, the lack of phenotypic information forms a major obstacle for the educated choice of genebank accessions for research and breeding. A promising approach to fill this information gap is to exploit historical information gathered routinely during seed regeneration cycles. Still, this data is characterized by a high non-orthogonality hampering their analysis. By examining historical data records for flowering time, plant height, and thousand grain weight collected during 70 years of regeneration of 6,207 winter wheat (*Triticum aestivum* L.) accessions at the German Federal *ex situ* Genebank, we aimed to elaborate a strategy to analyze and validate non-orthogonal historical data in order to charge genebank information platforms with high quality ready-to-use phenotypic information. First, a three-step quality control assessment considering the plausibility of trait values and a standard as well as a weather parameter index based outlier detection was implemented, resulting in heritability estimates above 0.90 for all three traits. Then, the data was analyzed by estimating best linear unbiased estimations (BLUEs) applying a linear mixed-model approach. An *in silico* resampling study mimicking different missing data patterns revealed that accessions should be regenerated in a random fashion and not blocked by origin or acquisition date in order to minimize estimation biases in historical data sets. Validation data was obtained from multi-environmental orthogonal field trials considering a random subsample of 3,083 accessions. Correlations above 0.84 between BLUEs estimated for historical data and validation trials outperformed previous approaches and confirmed the robustness of our strategy as well as the high quality of the historical data. The results indicate that the IPK winter wheat collection reveals an extraordinary high phenotypic diversity compared to other collections. The quality checked ready-to-use phenotypic information resulting from this study is the first brick to extend traditional, conservation driven genebanks into bio-digital resource centers.

## Introduction

Plant breeders improve the performance of crops by means of controlled crosses followed by selection (Bernardo, 2010; Becker, 2011). The first step intends to maximize diversity for those traits aimed to be improved, which in turn allows plant breeders to find those progenies that enhance the desired trait levels. Consequently, the genetic improvement of crops relies on the genetic diversity existing within plant populations. In line breeding of species like wheat, the parental material used in crosses can be of many different types (National Research Council, 1993; Sachs, 2009; Hammer and Knüpffer, 2015). This ranges from very well adapted material like released varieties or advanced breeding lines to less adapted old varieties, landraces, and even crop wild relatives. Nevertheless, most breeders have been reluctant to use less adapted plant material to perform crosses due to the potential risk of disrupting haplotype blocks associated to high crop performance that are present in elite material (National Research Council, 1993).

The evolution of *ex situ* conservation – in general known as genebanks – was divided into four major time eras by Cohen et al. (1991), where (i) in the “Era of plant exploration and introduction (1850 to 1950)” accessions were studied taxonomically and their utility was tested, (ii) in the “Era of conservation (mid 1950s to 1980s)” a wide spectrum of diversity was conserved, induced by the adoption of high-yielding varieties and the displacement of local varieties, (iii) in the “Era of regeneration and new international linkages (1990s)” the long-term viability of the collections were ensured in combination with international agreements and cooperation and (iv) in the “Era of more efficient utilization (2010 and beyond)” there is an enhanced exploitation of genetic resources by breeding. In order to guard against genetic erosion due to the recurrent use of elite material in crosses, genebanks are expected to be a rich source of alleles that are absent in breeding programs. Moreover, these alternative sources of trait variation and adaptation would be of uppermost importance to confront the challenging future scenario imposed by climate change and the continuously growing world population (Ray et al., 2012; Hall and Richards, 2013).

Maintenance methods for germplasm collections in wheat can be classified into two types: *in situ* and *ex situ* (Hammer and Knüpffer, 2015). *In situ* maintenance denotes the preservation of plant material at their native habitats, e.g., landraces and wild wheats on farms and geographic areas of high genetic diversity, respectively. In contrast, *ex situ* maintenance is the preservation of plant material outside of its original agro-ecological context. However, in both kinds of maintenance methods, seed regeneration is essential both to provide seeds to external users and to maintain germination capacity. In many *ex situ* collections, curators monitor the identity and purity of individual accessions, by collecting characterization and evaluation data during each regeneration cycle (Oppermann et al., 2015). However, data gathered during seed regeneration cycles is characterized by a high non-orthogonality. This is due to the fact that every year only a small proportion of the total collection is cultivated in unreplicated plots with changing regeneration frequency across the years. Therefore the unbiased analysis of historical data while keeping a maximum of their information content is challenging.

The German Federal *ex situ* Genebank of agricultural and horticultural crops maintained by the Leibniz Institute of Plant Genetics and Crop Plant Research (IPK) in Gatersleben is the largest collection in the European Union and ranks among the ten largest collections worldwide (Oppermann et al., 2015). The genebank is preserving 151,000 accessions comprising close to 3,000 plant species of 756 genera. Every year about 8,000 accessions are regenerated to keep up viability of seeds and to comply with 600 user’s requests involving approximately 30,000 samples. The IPK collection comprises 27,350 accessions of the genus *Triticum*. Comprehensive historical characterization and evaluation data are available for 6,207 winter wheat accessions (*Triticum aestivum* L). The information hidden in these historical data is of high value for all potential genebank users. In this context, first analysis of historical data allowed for discovery of new alleles for flowering time by means of targeted allele mining (Keilwagen et al., 2014).

The aim of this study was to elaborate a strategy to analyze a non-orthogonal historical data set and validate their quality and usefulness for the selection of accessions with defined trait performance characteristics. In particular our objectives were to (i) implement a three-step strategy for data quality assessment comprising a plausibility check, a standard outlier detection method and a weather parameter index based outlier detection, (ii) study the role of the missing value structure on the precision to estimate variance components and genotypic values, (iii) validate parts of the historical data in multi-environmental, orthogonal field trials, (iv) examine the phenotypic diversity of the IPK winter wheat collection and their relevance for plant breeding and research, and (v) give recommendation on genebank regeneration strategies to warrant the usability of the collected data.

## Materials and Methods

### Plant Material

The present study included 6,207 accessions of winter wheat (*T. aestivum* L.) from the German Federal *ex situ* Genebank of agricultural and horticultural crops maintained at the IPK Gatersleben. Sixty-six percent of the accessions were collected in Europe and the countries of the former Soviet Union. Thereof, approximately one third of the wheat accessions originate from Germany (11%), Italy (11%), and countries of the former Soviet Union (10%). For 10% of the accessions the origin was unknown. Around 13% of the accessions originate from the Middle East and 6% were collected in the Far East. Notable collections covered also North America (6%).

### Field Trials of the Historical Data

**FIGURE 1:**
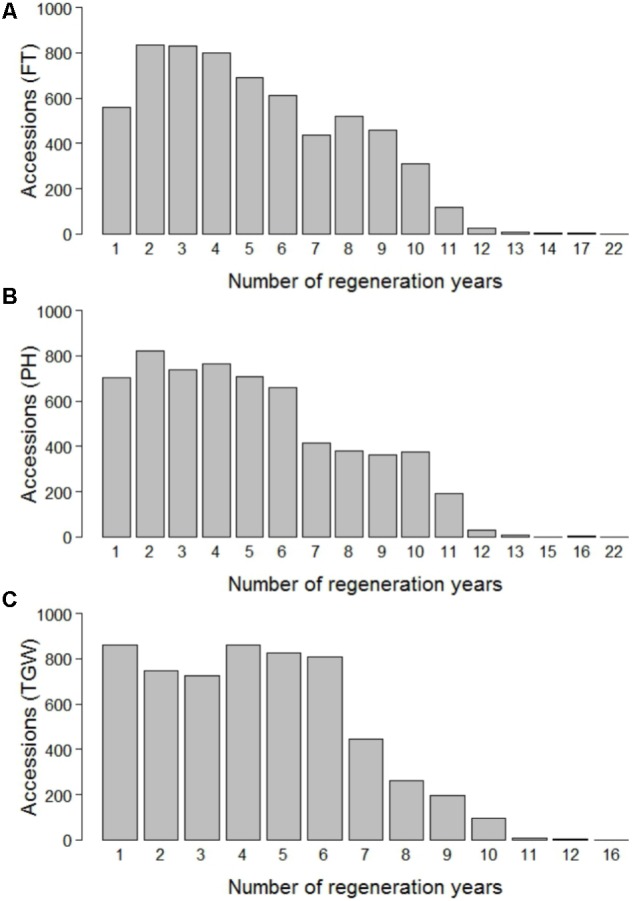
Number of regeneration years per accession for flowering time (FT) **(A)**, plant height (PH) **(B)**, and thousand grain weight (TGW) **(C)**.

The phenotypic data was routinely collected in the course of the seed regeneration activities. Amongst others, the accessions of winter wheat were evaluated for flowering time (FT), plant height (PH), and thousand grain weight (TGW) between harvest season 1946 and 2015 in Gatersleben, Germany (latitude 51° 49′ 19.74″ N, longitude 11° 17′ 11.80″ E, 110.5 m.a.s.l., black soil of clayey loam type, 9°C average annual temperature, 490 mm average annual rainfall). The evaluation in unreplicated trials was based on plots with a size of 3.75 m^2^. A randomized experimental design was assumed. FT corresponds to the date on which plants reached stage Z65 (Zadoks et al., 1974) and is expressed as days after January 1st. PH was measured as the distance between soil surface and the top of the spike (including awns) at grain filling (growth stage Z70, Zadoks et al., 1974) and is expressed in cm. TGW was determined in accordance with the official seed board regulations and is expressed as the weight of thousand grains in g. For FT, PH, and TGW, observations were made in 69, 70, and 59 different years and 31,817, 31,139, and 25,808 phenotypic records were available for 6,206, 6,164, and 5,841 accessions, respectively. Until 1975, each accession was regenerated and evaluated in time intervals of maximum 4 years (Supplementary Figure S1). Cold storage was implemented in 1976, which allowed for an extension of regeneration intervals to up to 30 years. On average 452, 439, and 439 accessions were phenotyped per year for FT, PH, and TGW, respectively, with a peak of 3,008 accessions in the year 1970 and a minimum of 37 accessions in the year 2014 for FT. Each accession was evaluated on average 5.03, 4.99, and 4.29 times for FT, PH, and TGW, respectively, again with substantial variation across years (**Figure 1**).

### Phenotypic Data Analyses of the Historical Data

Before analyzing the data we implemented a plausibility check on historical data guaranteeing that only autumn sown hexaploid winter wheat accessions with physiologically possible trait records and publically available accessions were entering the analysis. Details on the plausibility check are described in Supplementary Table S1.

Then we fitted the following linear mixed model to analyze the historical data of each trait:

(1) $$\begin{array}{c} y_{\mathrm{ij}}=\mu +\;g_{i}+\;a_{j}+\;e_{\mathrm{ij}} \end{array}$$

where *y*_ij_ referred to the phenotypic value of the *i*th accession in *j*th year, μ was the intercept, *g*_i_ was the effect of the *i*th accession, *a*_j_ referred to the effect of *j*th year, and *e*_ij_ were the residuals. The variances of the residuals were assumed to be heterogeneous across years. For testing outliers, we used studentized residuals following the proposal of Nobre and Singer (2011). The Bonferroni–Holm method was applied to correct for multiple testing (Holm, 1979). All detected outliers were removed and Equation [1] was again fitted. Variance components estimated before and after outlier detection were compared. In Equation [1], we assumed random year and fixed accession effects for outlier detection and to compute best linear unbiased estimations (BLUEs) of accessions. Phenotypic variance components were estimated assuming accession and year effects as random. Later, heritabilities were calculated by the following formula:

(2) $$\begin{array}{c} h^{2}=\frac{\sigma_{G}^{2}}{\sigma_{G}^{2}+\frac{\sigma_{e}^{2}}{Year}} \end{array}$$

where $\sigma_{G}^{2}$ refers to the variance of accessions, $\sigma_{e}^{2}$ to the average variance of residuals and *Year* to the average number of years each accession was tested.

### Weather Data and Weather Parameter Index

Comprehensive monthly weather records were available at the experimental site for rainfall, air humidity and temperature (average, T.avg; minimum, T.min; maximum, T.max) measured 2 m above the ground for the time span between 1953 and 2015. Between April 1993 and November 1999 no records for T.min and T.max were available. Stepwise multiple regressions were used to identify standardized weather parameters explaining the highest proportion of the variation for year effects (YE) on each trait as estimated with Equation [1] assuming random genotype and fixed year effects. The resulting regression coefficients were used to define an aggregated weather parameter index for each trait × year combination. This index was compared to the coefficient of variation (CV) of the year specific error variances calculated as:

(3) $$\begin{array}{c} CV=\frac{\sqrt{\sigma_{\mathrm{e*}}^{2}}}{YE} \end{array}$$

where $\sigma_{\mathrm{e*}}^{2}$ refers to the year specific error variance and YE to the corresponding year effect. An inflated CV in combination with weather parameter index anomalies indicates that unfavorable weality of phenotypic data in a specific regeneration year.

### Validation of the Results of the Historical Data in Replicated Multi-Environmental Trials

The validation experiments included a random sample of 3,083 genebank accessions and 47 advanced elite lines of current agronomical relevance in Germany. Together they were evaluated for FT and PH in two different trials in up to five environments in Gatersleben and Schackstedt (latitude 51° 43′ 0″ N, longitude 11° 37′ 0″ E, 122.0 m.a.s.l., black soil of clayey loam soil type, 8.9°C average annual temperature, 483 mm average annual rainfall) during harvest years 2014, 2015, and 2016. In each trial × environment combination accessions were randomized following an alpha lattice experimental design with a plot size of 0.4 m^2^. Each plot consists of a double-row with 100 plants in total. The trials were connected by up to 15 common checks.

We analyzed the validation experiments by fitting following linear mixed model for each trait:

(4) $$\begin{array}{c} y_{\mathrm{ijklm}}=\mu +\;g_{i}+v_{j}+{\left(gv\right)}_{\mathrm{ij}}+t_{k}+\;r_{l\left(k\right)}+\;b_{m\left(\mathrm{kl}\right)}+\;e_{\mathrm{ijklm}} \end{array}$$

where _y_ijklm__ was the phenotypic value of the *i*th accession in the *j*th environment in the *k*th trial in the *l*th replication within the *m*th block, μ was the intercept, *g*i referred to the effect of the *i*th accession, *v*_j_ was the effect of *j*th environment, (*gv*)_ij_ was the interaction of *i*th accession with the *j*th environment, *t*_k_ the effect of *k*th trial, *r*_l_(k) the effect of *l*th replication in *k*th trial, b_m_(*kl*) the effect of *m*th block in *l*th replication within *k*th trial, and e_ijklm_was the residual of y_ijklm_. Accessions were set as fixed effects and all others were considered as random effects. Linear mixed models were solved using the restricted maximum likelihood (REML) algorithm as implemented in the ASReml-R package (Butler et al., 2009).

Correlations between BLUEs of validation experiments and the historical data were calculated considering 2,232 and 2,221 overlapping accessions for FT and PH, respectively. Finally, BLUEs of accessions evaluated in the validation experiments were also correlated with estimations from the historical data reported by Keilwagen et al. (2014). Specifically, these authors reported arithmetic means and normalized rank product (NRP) for each accession.

### Impact of Deviations From the Assumption of Missing at Random

The historical data is characterized by a high degree of missing values, because only 7% of the total number of analyzed accessions has been regenerated on average in each year. Before the implementation of the cold storage facilities, regeneration was performed in a block-wise manner, which was mainly based on the year the accessions entered the genebank. Moreover, blocks sometimes reflect collection hotspots of certain years. This was for instance the case for 391 accessions originating from Iran from which 91% were first regenerated in the year 1960 (Supplementary Figure S2). One basic assumption of the REML algorithm on non-orthogonal data is that the missing data follows either a missing-completely-at-random (MCAR) or a missing-at-random (MAR) pattern (Piepho and Möhring, 2006). The block-wise regeneration may deviate from the MAR assumption when blocks are not composed by random samples of accessions. We therefore performed a resampling study to investigate the potential bias in estimating first and second-degree statistics caused by block-wise missing value structures. For this, we sampled a balanced sub data set of 160 accessions, which were all evaluated for FT and PH in the years 1951, 1953, 1956, 1959, 1964, and 1970. These 160 accessions originate from 8 countries: 51 from Germany, 42 from the United States, 27 from Sweden, 15 from Greece, 7 from France, 6 from Afghanistan, 6 from Albania, and 6 from Great Britain. Based on the 160 accessions a resampling study was performed mimicking block structures resulting due to common geographic origins (Scenario A; Supplementary Figure S3A) or year of collection (Scenario B; Supplementary Figure S3B). In addition, a MCAR scenario was implemented (Scenario C; Supplementary Figure S3C). For Scenario A, we used the eight different geographic origins for blocking and assumed that every block was evaluated in three out of the 6 years. For Scenario B, the same block sizes and phenotyping intensity were assumed as in Scenario A but the geographic origin of the accessions was ignored. For Scenario C, we ignored blocks and assumed the same phenotyping intensity per accession as for the Scenarios A and B. Resampling was repeated 100 times and the BLUEs as well as the phenotypic variance components were estimated in each resampling run. All computational methods were implemented in R environment (R Core Team, 2016).

## Results

### Three-Step Strategy to Assess the Quality of Phenotypic Data Resulted in Increased Heritability Estimates

**FIGURE 2:**
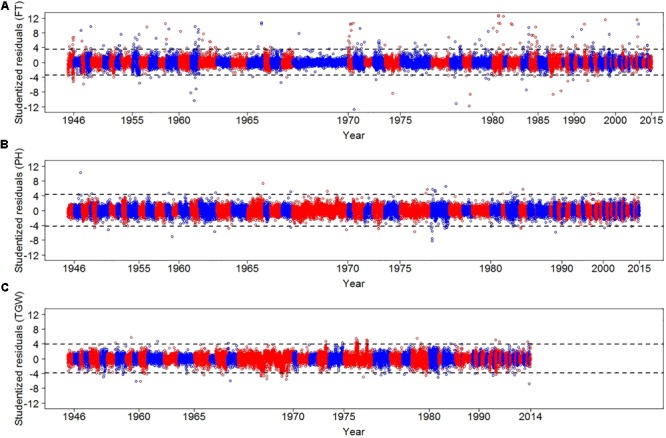
Studentized residuals of FT **(A)**, PH **(B)**, and TGW **(C)** for each regeneration year. Residuals were estimated by modeling independent variances of residuals for each of the regeneration years. We alternated between red and blue colors to distinguish consecutive years. Dashed lines display thresholds for defining outliers. For TGW the outlier year 1961 is not shown. Please note that due to different data density per year and years without data the *x*-axes are not linear.

We implemented a three-step strategy to assess the quality of the historical phenotypic data. First, plausibility checks were performed and data from other species, spring wheat, no longer existing accessions, and records with unusual sowing time or physiological impossible trait observations were removed (see Supplementary Table S1 for more details). Second, we implemented a standard outlier detection method. Third, we used historical weather data to identify years with inflated CV of the residuals. In total 31,817, 31,139, and 25,808 data points survived the plausibility tests for FT, PH, and TGW, respectively, and underwent outlier detection, where we identified 251, 46, and 93 outliers for FT, PH, and TGW, respectively (**Figure 2**). Outliers were excluded from the dataset and corresponded to 0.79, 0.15, and 0.36% of FT, PH, and TGW records, respectively.

The CV of the residuals was inspected for each trait × year combination (Supplementary Figure S4). We detected an inflated CV of the residuals for TGW in the year 1961, which was 2.82 times larger compared with the average CV of the other years. To verify whether extreme weather events occurred in the year 1961, a stepwise multiple linear regression was performed using BLUEs of year effects for TGW as dependent variable and monthly recorded rainfall, air humidity, as well as T.avg, T.min, and T.max as explanatory variables. For model selection, GLMSELECT procedure of the SAS system (SAS Institute Inc, 2011) was applied. Maximum temperature in February, rainfall in April and June as well as air humidity in April were selected explaining 38% of the variation of the year effects of TGW. The selected variables were combined to a weather parameter index, which revealed for 1961 an extreme value, which was 4.14 times lower than the average negative index values across years. In particular, rainfall was 3.6 higher in April 1961 than on average in the other years (Supplementary Figure S5). This high rainfall during 1961 could have caused severe infections due to Septoria leaf blotch which is spread by rain splash to the upper leaves (Shaw and Royle, 1993) detrimentally affecting TGW (Shaw and Royle, 1989) in an extraordinary manner. Consequently, we excluded all 600 records of TGW for the year 1961 for further analysis.

The implemented three-step strategy to perform quality assessment had a substantial impact on the variance of the residuals, which decreased by 29, 4, and 15%, for FT, PH, and TGW, respectively (**Table 1**). In contrast, the genetic variances changed only marginally for the three traits with differences ranging from 1% for PH to 3% for FT. Heritability estimates increased by a maximum of 3% for FT and were high for all traits with values above 0.90.

**Table 1:** Second-degree statistics for flowering time (FT), plant height (PH), and thousand grain weight (TGW) before and after outlier correction, where $\sigma_{Y}^{2}$ refers to the variance of the years, $\sigma_{G}^{2}$ denotes the genetic variance, $\sigma_{e}^{2}$ is the variance of the residuals, Number of years is the average number of years when accessions were regenerated, and h^2^ refers to the heritability.

Source	FT	PH	TGW
**Before outlier correction**
$\sigma_{Y}^{2}$	72.36^∗∗∗^	228.75^∗∗∗^	17.91^∗∗∗^
$\sigma_{G}^{2}$	15.23^∗∗∗^	342.15^∗∗∗^	28.98^∗∗∗^
$\sigma_{e}^{2}$	9.08	102.62	16.13
Number of years	5.13	5.05	4.42
h^2^	0.90	0.94	0.89
**After outlier correction**
$\sigma_{Y}^{2}$	71.95^∗∗∗^	230.99^∗∗∗^	15.02^∗∗∗^
$\sigma_{G}^{2}$	15.62^∗∗∗^	346.72^∗∗∗^	29.67^∗∗∗^
$\sigma_{e}^{2}$	6.48	98.53	13.76
Number of years	5.09	5.04	4.30
h^2^	0.92	0.95	0.90

*^∗∗∗^P < 0.001.*

### Block-Wise Missing Value Structure Can Influence Precision in Estimating First- and Second-Degree Statistics When Analyzing Historical Genebank Data

**FIGURE 3:**
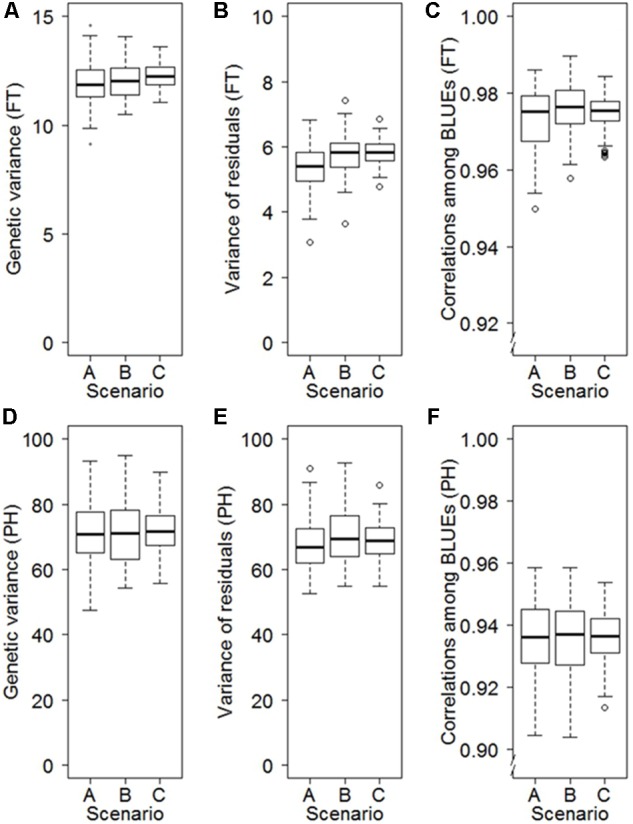
Influence of different patterns of missing values on the precision in estimating the genetic variance **(A)**, the variance of the residuals **(B)** and best linear unbiased estimations (BLUEs) **(C)** for FT as well as the genetic variance **(D)**, the variance of the residuals **(E)** and BLUEs **(F)** for PH. The scenarios are based on 100-fold repeated sampling of historical data from 3 years from an orthogonal dataset including 160 accessions evaluated in 6 years. Patterns of missing values are based on eight blocks of accessions defined according to their origin (Scenario A), eight blocks of accessions of the same size as Scenario A but with random origin (Scenario B), and ignoring any block structure (Scenario C).

The data collected during annual seed regeneration until establishment of the cold storage facilities in 1976 followed a block-like missing value structure according to the years of collection (Supplementary Figure S1), which is often associated with the geographic origin of accessions (Supplementary Figure S2). The geographic origins can influence phenology traits (Langer et al., 2014; Würschum et al., 2015). We therefore investigated the risk of obtaining biased estimates of first- and second-degree statistics when analyzing historical data generated during genebank regeneration by applying a resampling approach. The resampling study revealed that the block-like missing value structure led to only a minor average bias, which ranged between – 0.04 and 0.05% depending on the trait (**Figure 3**). The standard deviations of 100 resampling runs were, however, much smaller for a random scenario (Scenario C) than for the studied block-like missing value structures (A and B Scenarios). For example the standard deviations of the point estimates for the genetic variance of FT were elevated by 57% for Scenario B and 105% for Scenario A compared to Scenario C (**Figure 3**).

### Validation of the Results of the Historical Data Sets Based on Orthogonal Field Trials

**FIGURE 4:**
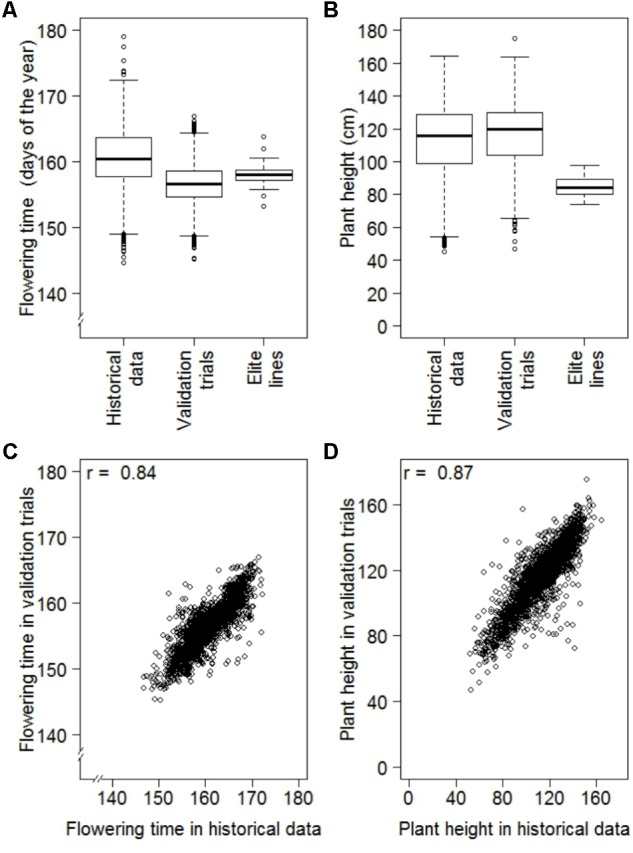
Comparison of best linear unbiased estimates (BLUEs) of historical data with designed replicated multi-environmental validation trials consisting of a random sample of genebank accessions (Validation trials) and German advanced elite lines, where **(A)** are the distributions of BLUEs estimated for flowering time of 6,206 accessions of historical data, 2,232 accessions of the validation trials and 47 advanced elite lines, **(B)** are the distributions of BLUEs estimated for plant height of 6,164 accessions of historical data, 2,221 accessions of the validation trials and 47 advanced elite lines, **(C)** is the correlation between 2,232 common accessions of the historical data and validation trials for flowering time and **(D)** is the correlation between 2,221 common accessions of the historical data and validation trials for plant height.

The results of the historical data sets were validated performing designed multi-environmental trials focused on PH and FT. Data quality of validation trials was high with heritabilities of 0.95 and 0.85 for PH and FT, respectively. For both traits, the ranges of BLUEs from validation experiments were comparable to those portrayed in the historical data (**Figure 4**). The mean of FT observed in the validation trial, however, was 4.30 days lower than that for the historical data (**Figure 4**). This difference can be explained by a temporal trend toward earlier flowering across the last 70 years (Supplementary Figure S6). The correlation of BLUEs between the historical data and validation experiment were high with a coefficient of 0.84 for FT and 0.87 for PH (**Table 2**). Moreover, we contrasted our analyses strategy with one based on arithmetic means and NRP estimated in a previous study (Keilwagen et al., 2014). For both traits, we observed a higher correlation between BLUEs estimated with the historical data and validation trials as compared to those between arithmetic means or NRP of the historical data reported by Keilwagen et al. (2014) and the validation trials (**Table 2**).

**Table 2:** Pearson’s correlation coefficients among best linear unbiased estimations of validation trials (BLUES_Validation_) and best linear unbiased estimations of the historical data (BLUES_Historical_), the arithmetic means of the historical data (Mean_Historical_), and the normalized rank product of the historical data (NRP_Historical_) for flowering time (FT) and plant height (PH).

Source	BLUES_Validation_	
	FT	PH
BLUES_Historical_	0.84^∗∗∗^	0.87^∗∗∗^
Mean_Historical_	0.70^∗∗∗^	0.83^∗∗∗^
NRP_Historical_	0.76^∗∗∗^	0.76^∗∗∗^

*^∗∗∗^P < 0.001. Mean_*Historical*_ and NRP_*Historical*_ were previously published by Keilwagen et al. (2014). The correlations of flowering time and plant height were estimated based on 2,232 and 2,221 common accessions, respectively*.

### Broad Phenotypic Diversity Observed in the Winter Wheat Collection of the IPK Gatersleben

**FIGURE 5:**
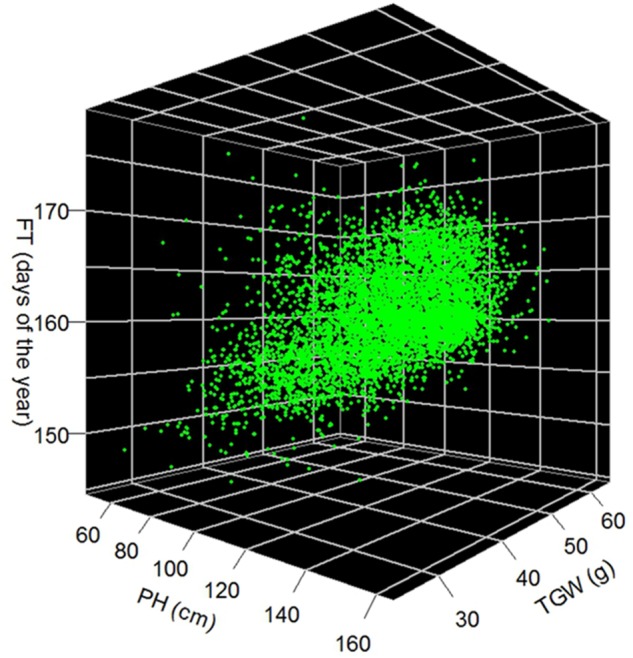
Best linear unbiased estimations of 5,799 winter wheat accessions for FT in days of the year, PH in cm and TGW in g.

We observed a moderate (*r* = 0.51; *P*-value < 0.001) correlation between FT and PH, while correlation between FT and TGW as well as PH and TGW were much less pronounced with coefficients of 0.05 (*P* < 0.001) and 0.16 (*P* < 0.001), respectively (**Figure 5**). The estimates of FT of the historical data varied between 144.6 and 179.1 days of the year, which corresponds to a period between the 24th of Mai and the 28th of June. The average FT was the 160.6th day of the year. Comparing the FT of the historical data with the validation trials the genetic variance of historical data was overestimated by 14%, while the genetic variance of the advanced elite lines was 86% lower compared to the validation trials (**Figure 4A**). The BLUEs for PH of the historical data varied between 45.3 and 164.5 cm. The average PH was 113.0 cm. Comparing the PH of the historical data with the validation trials, the genetic variance of historical data was underestimated by 14%, while the genetic variance of the advanced elite lines was 95% lower compared to the validation trials (**Figure 4B**). Last but not least, the estimates for TGW of the historical data varied between 24.3 and 65.7 g with an average of 46.9 g.

## Discussion

### Charging the Genebank Information Platforms With Phenotypic Data Is Challenging

For feeding the future McCouch et al. (2013) proposed to mine biodiversity of genebanks in three steps to overcome future food shortages. Briefly, this includes (i) the genetic fingerprinting of the accessions, (ii) phenotyping of the accessions, and (iii) the presentation of the data in an internationally accessible informatics infrastructure, where the phenotyping is considered as “…the most intellectually challenging, complex, costly and time-consuming stage” (McCouch et al., 2013).

In order to overcome the gap of phenotypic information of genetic resources Keilwagen et al. (2014) suggested making historical data produced during seed regeneration of genebank accessions accessible by applying a NRP approach. Obviously, this approach is limited since rank values of the accessions range between 0 and 1. Hence, the information system of the IPK’s Genebank (Genebank Information System, GBIS) currently provides solely the passport data which includes, for example, information about taxonomy, collection site and collection time (Oppermann et al., 2015). Thus, due to the lack of phenotypic information, the educated choice of accessions for research and breeding is still limited. Evidently this problem is also faced by information systems developed to access data of genebank collections at the European (Weise et al., 2017) or worldwide level (GENESYS, 2018). Therefore, the challenge of each single genebank worldwide is to provide quality checked adjusted entry means of their accessions in a standardized, comparable fashion. Against this backdrop, we elaborated and validated a strategy to analyze non-orthogonal historical regeneration data in order to charge genebank information platforms with ready-to-use phenotypic information.

### Valuable Phenotypic Data as Side Product of Genebank Management

At the IPK genebank the regeneration pattern of the wheat accessions analyzed in this study changed over time. While until 1975 each wheat accession was regenerated every 2–4 years in a block-wise manner together with other accessions entering the genebank at the same time, the implementation of cold storage facilities in 1976 enlarged the regeneration interval per accession by up to three decades (Supplementary Figure S1). Nonetheless, when the data is filtered by regeneration pattern, the block-wise strategy results in relatively dense data with many evaluation years per accession but the risk of potentially biased estimates, while the random pattern from 1976 onwards fulfills the requirements for an unbiased analysis with the REML-approach but provides much less evaluation years per accession (**Figure 3** and Supplementary Figure S1). Together the data forms a highly non-orthogonal data set with at least 93% of missing data for the full accession × year matrix depending on the trait under consideration. Additionally, temporal trends have to be considered. Accessions tend to flower 0.27 days earlier per year across the last 70 years (Supplementary Figure S6) while the phenotyping protocols did not change substantially.

### Rigorous Quality Management Pays off When Analyzing Historical Data

The proposed strategy of analyzing historical data has a clear focus on quality assessment. In our three-step approach the plausibility test plays an essential role requiring sufficient knowledge about taxonomy, physiology, and agricultural practice which is often ignored in pure statistical analysis. However, while standard outlier detection methods may detect physiologically unlikely records of the trait, the model cannot distinguish among, e.g., different species or season and off-season sowing in the data set, which potentially will bias the results.

The second step is the statistical outlier detection. The applied approach in particular seems to play a minor role and we implemented for the non-orthogonal data structure a Bonferroni–Holm test in combination with studentized residuals (Nobre and Singer, 2011; Bernal-Vasquez et al., 2016). For FT the outlier test was reducing the error variance by up to 29% while keeping the genetic variance approximately constant compared to the analysis without outlier detection (**Table 1**). This was achieved by omitting only 0.79% of the data as outlier, which is quite efficient.

The last step is the outlier detection relying on historical weather data. While the first two quality checks were record-based approaches, the last step is an environment-based outlier detection. Weather conditions are influencing the development of plant growth. Especially the epidemic spread of diseases like Septoria leaf blotch that in particular influences yield performance of the plants can be forecasted by weather parameters (e.g., Henze et al., 2007; te Beest et al., 2009; El Jarroudi et al., 2017). If the CV of the error variance of a particular environment is inflated and this inflation could be explained by weather parameters we suggest excluding all records for this trait from this outlier environment in order to improve data quality. Heritability estimates above 0.9 for all traits confirm the utility of the three-step quality control.

### Linear Mixed Model Approach Estimating BLUEs Is Advantageous

Orthogonal data validation outperformed the arithmetic means and the NRP-approach published by Keilwagen et al. (2014) (**Table 2**). Additionally, the BLUEs expressed in metric units for time (FT in days), height (PH in cm), and weight (TGW in g) have the advantage to be easily comparable across studies and populations in contrast to NRP-values. The interpretation of the latter is contextually limited, e.g., the position of an accession or cultivar from outside the collection with a plant height of 100 cm can easily be classified among ordered BLUEs ranging from 45.3 to 164.5 cm, while it is not possible to classify 100 cm into rank-values ranging between zero and one.

In order to analyze non-orthogonal data sets Piepho and Möhring (2006) suggested preferring best linear unbiased predictions (BLUPs), obtained by assuming the effect of the accession also as random in Equation [1], to BLUEs. The main difference between both would be that the BLUP-approach might change the rank of the accessions due to genotype specific shrinkage toward the mean value. However, in the historical data the Pearson correlation and the Spearman rank correlation between BLUEs or BLUPs and the validation trials were almost identical for both traits. A further reason why we prefer the estimation of BLUEs is that BLUPs will end up in a double shrinkage of the accessions when historical data is used later for genome-wide association mapping or genomic prediction.

### Structure of Missing Pattern Has an Influence on the Precision of Estimates of First and Second Decree Statistics

In general three missing pattern can be distinguished (i) missing-completely-at-random (MCAR), (ii) missing-at-random (MAR) and (iii) not-missing-at-random (NMAR), where MCAR is fulfilled if the missing value pattern does not depend on the values of missing or observed data, MAR is fulfilled if the missing value pattern only depends on the observed values and not on the missing values and NMAR is fulfilled if the missing value pattern depends on the missing values in the data (Little and Rubin, 2002). For unbiased estimates in analyzing non-orthogonal data by a REML approach the drop-out pattern has to fulfill the MCAR or the MAR criteria as discussed by Piepho and Möhring (2006). While the block-wise regeneration of the historical data until 1975 refers to a MAR pattern simulated in Scenario A and Scenario B, the regeneration structure beginning from the introduction of cold storage facilities in 1976 fulfills the criteria of MCAR simulated in Scenario C (**Figure 3**). As expected, the overall means of the validation runs in the simulation study did not differ substantially for the estimations of the variance components and BLUEs. However, the MCAR Scenario C had highest precision in estimating these parameters in contrast to the MAR scenarios. From the latter, Scenario A, where the regeneration blocks were defined by the common origin of the accession, precision tend to be lower compared to Scenario B where accessions of each block were assembled randomly. One plausible explanation for this is that accessions differ in their trait performance by origin (Langer et al., 2014; Würschum et al., 2015), which is enlarging the variance between the blocks in Scenario A compared to Scenario B with random accessions per block, lowering the precision of the overall estimates. Therefore, in order to make best use of regeneration data we recommend that genebank curators regenerate the accessions following a MCAR pattern (Scenario C) where accessions for regeneration were not selected by origin or acquisition date. In genebanks it is common practice that accessions were regenerated when germination capacity or the seed stock dropped below a certain threshold, which indeed is a kind of selection. However, since seed request or germination capacity of the accessions is not correlated to the trait performance itself this is not violating the MCAR assumption. Additionally, the use of 5–10 common checks across a long term period would support the data analysis and further boost the data quality.

### Independent Validation of Historical Data Confirms Robustness of the Applied Strategy

The FT interval of 34.52 days of the historical data has to be interpreted carefully. As we can see from **Figure 4A**, a representative part of the data of 2,236 accessions was validated in orthogonal, multi-environmental field trials. In these validation trials we observed only an interval of 21.70 days for FT. While the minimum values for FT of the historical data and the validation trials were comparable with the 144.6th and 144.2th day of the year, respectively, the maximum values differed significantly by 12.3 days between the 179.1th day of the year for historical data and the 166.8th day of the year for validation trials. This last difference could be explained by the contrasting evaluation environments. The historical data were evaluated in 69 different years at the same location compared to the validation trials tested in three different years at two locations. In the historical data we observed a temporal trend of 0.27 days per year of early flowering (Supplementary Figure S6). Due to non-overlapping environments between the two data sets it is likely that FT values of current validation trials were shifted toward early flowering compared to the historical data. This was not only affecting the maximum values but also the average value decreased by 4.2 days. Additionally, some of the extreme performing accessions in historical data have low individual phenotyping intensity, influencing the precision of these estimations. Nevertheless, comparing the upper and lower 2.5% of the ranked accessions of historical data and validation trials, 57 and 43% of the minimum and maximum accessions overlapped, respectively. Next to the total correlation of 0.84 for FT of the two data sets, this is confirming the high quality of the historical data.

The range of the PH of the historical data was 119.2 cm and again a representative part of 2,221 accessions was used for validation (**Figure 4B**). Ignoring one outstanding tall accession of the validation trials (TRI 8278), descriptive statistics of historical data and validation trials were comparable with minimum values of 45.4 and 47.1 cm, maximum values of 164.5 and 163.7 cm, average values of 113.0 and 116.6 cm as well as ranges of 119.2 and 116.6 cm, respectively. Comparing the upper and lower 2.5% of the ranked accessions of historical data and validation trials, 59 and 46% of the minimum and maximum accessions overlapped, respectively. Next to the total correlation of 0.87 of the two data sets, this is also confirming the quality of historical data for PH.

### Exceptionally High Phenotypic Diversity of the IPK Winter Wheat Collection

While many studies focused on the genetic diversity of wheat as revealed by molecular markers in European (e.g., Würschum et al., 2013; Nielsen et al., 2014) and global (e.g., Cavanagh et al., 2013; Wang et al., 2014; Bonman et al., 2015) panels, only few studies investigated the phenotypic diversity of agronomic traits like FT (Langer et al., 2014; Zanke C. et al., 2014), PH (Zanke C.D. et al., 2014; Würschum et al., 2015), and TGW (Zanke et al., 2015) in European hexaploid winter wheat. Studies for hexaploid winter wheat diversity panels of worldwide origin on useful phenotypic traits are also very rare (Neumann et al., 2011; Wilhelm et al., 2013). However, knowledge about the physiologically possible ranges of these phenotypic traits plays a key role for breeders and researches for utilization of plant genetic resources and to cope with the future problems like the food shortage and climatic change (Lopes et al., 2015). The historical phenotypic data of the IPK winter wheat collection with 6,207 accessions collected or obtained from at least 100 present and former countries during the last 70 years is, to the best of our knowledge, the world–wide largest winter wheat collection investigated for these three traits.

In order to compare the phenotypic diversity of the IPK winter wheat collection with other collections we contrasted it to (i) a sample of 47 German advanced elite winter wheat lines investigated for FT and PH tested together with the validation trials, (ii) a European panel of 358 winter wheat and 14 spring wheat varieties, sampled across 13 European countries evaluated for the FT related trait heading date (Zanke C. et al., 2014), PH (Zanke C.D. et al., 2014), and TGW (Zanke et al., 2015) in eight environments and (iii) a world-wide collection of 96 diverse winter wheat accessions, sampled across 21 countries evaluated for FT, PH, and TGW in up to eight environments (Neumann et al., 2011) (Supplementary Table S2).

The genetic variance is less sensitive to extreme values within the raw data and therefore a good measure to compare diversity. The German advanced elite lines only represent 13 and 6% of the genetic variance which is present in the IPK winter wheat collection for FT and PH, respectively. As expected, the European panel was more diverse representing 63, 23, and 39% of the genetic variance existent for FT, PH, and TGW in the IPK collection. In contrast the world-wide panel of Neumann et al. (2011) outperformed the IPK collection representing 177, 111, and 133% of the genetic variance for FT, PH, and TGW of the IPK genebank. Nonetheless, this last observation must be carefully interpreted, because this relatively small world-wide collection was sampled from a bigger collection in order to maximize trait diversity (Neumann et al., 2011). Moreover, considering the ranges between the minimum and maximum phenotypic trait values this world-wide collection represents only 62, 84, and 74% of the ranges compared to the IPK collection for FT, PH, and TGW, respectively.

The high quality of the historical data of the IPK winter wheat collection confirmed by high heritability estimates above 0.90 (**Table 1**) and validation field trials reveals a competitive phenotypic diversity compared to European and even global collections. Nevertheless, since the collection hot spots of the IPK winter wheat collection were Europe and Asia, there is still potential to boost the phenotypic diversity by filling collection gaps using plant material from other geographic regions. For instance, major wheat growing countries like India and China as well as accessions from other continents in which winter wheat plays a minor role (e.g., South America, Africa, and Oceania) seem to be underrepresented in the collection. However, especially wheats from tropical and subtropical latitudes will lack in their environmental adaptation (e.g., frost tolerance, photoperiod sensitivity and disease resistances) when regenerated at the IPK genebank which is may masking their phenotypic performance. This is one of the limitations in *ex situ* conservation.

### Utilization of Historical Data for Plant Breeding and Research

The three agronomic traits investigated in this study are key factors for wheat breeding. While FT is crucial for the global and seasonal adaptation of wheat (Worland, 1996; Kamran et al., 2014), PH played a central role during the Green Revolution when semi-dwarfing genes were introduced into global breeding material (Hedden, 2003) significantly increasing lodging resistance, harvest index and grain yield of wheat (Borlaug, 1968; Austin et al., 1980; Börner et al., 1993; Flintham et al., 1997; Brancourt-Hulmel et al., 2003). Next to the number of spikes per area and kernels per spike TGW is considered as a major yield component (Simmonds et al., 2014). Due to its high heritability this indirect trait is of importance for yield related research in wheat (e.g., Groos et al., 2003; Cuthbert et al., 2008; Neumann et al., 2011; Lopes et al., 2012; Sukumaran et al., 2015). However, while the major loci of FT and PH (Distelfeld et al., 2009; Liu et al., 2012; Würschum et al., 2015) are relatively well understood, the genetic architecture of TGW is more complex (Liu et al., 2012; Nadolska-Orczyk et al., 2017). The high quality historical data of the IPK winter wheat collection provide a valuable source of phenotypic information for association studies mining loci and alleles which have a potential use in the fine adjustment of these traits.

At present, about 4,000 accessions of the IPK winter wheat collection still lack useful phenotypic information. With sufficient molecular marker coverage, the available historical data can be used for a genome-wide prediction, in order to completely charge the genebank with phenotypic information (Crossa et al., 2016; Yu et al., 2016).

Further, researchers will be able now to assemble their individual core collections or targeted panels based on phenotypic traits of 6,207 accessions, e.g., to avoid bias in plant developmental stage by too much variation in FT or PH when studying ear and leaf diseases under field conditions. In addition, breeders can also benefit from historical data. Based on FT estimates in days from the 1st January they can select accessions, which fit to their national breeding pools in terms of local adaptation or they use the data in order to synchronize FT of the accessions and elite material to uncover the breeding value of these accessions in a hybrid strategy (Longin and Reif, 2014). This targeted selection of potentially beneficial accessions based on provided phenotypic information is a cost and time effective way of utilizing the most promising accessions out of the unmanageable amount of plant genetic resources hosted by the genebank. The same goal has the Focused Identification of Germplasm Strategy (FIGS) which uses detailed information about the eco-geographic environment from which accessions were collected to predict where plant traits are likely to have evolved and thus have a high genetic variation of the considered trait (Sanders et al., 2013). However, to implement the FIGS algorithm a part of the genebank accessions have to be evaluated for the trait under consideration to train the prediction model. High quality historical data can be used to improve the training of these prediction models in order to select promising accessions without previous phenotypic records but information about their origin from genebanks.

The results of the present study form a substantial contribution to extend the IPK Genbank into a bio-digital resource center. The widespread use of wheat genetic resources will be spurred by the integration of the augmented phenotypic data generated by this study to further enhance its information and ordering system. Moreover, the integration of marker and sequence data will further boost the practical and the academic value of the wheat collection.

## Author Contributions

NP and JR designed the study. NP and AS wrote the manuscript. NP did the data analysis. SW, MO, AB, AG, JK, and BK provided the data and background information about the historical data and helped to improve the manuscript. YZ supported the data analysis. All authors helped to improve the manuscript.

## Conflict of Interest Statement

The authors declare that the research was conducted in the absence of any commercial or financial relationships that could be construed as a potential conflict of interest.
