# Supplementary material for: Leveraging the Use of Historical Data Gathered During Seed Regeneration of an ex Situ Genebank Collection of Wheat
Source: Front Plant Sci. 2018 May 8;9:609. doi: 10.3389/fpls.2018.00609 (PMC5953327; doi:10.3389/fpls.2018.00609)
Supplement: Supplementary file 1 [file Table_1.DOCX]

***Supplementary Material***

**Leveraging the use of historical data gathered during seed regeneration of an *ex situ* genebank collection of wheat**

**Norman Philipp^1^, Stephan Weise^2^, Markus Oppermann^2^, Andreas Börner^2^, Andreas Graner^2^, Jens Keilwagen^3^, Benjamin Kilian^4^, Yusheng Zhao^1^, Jochen C. Reif^1^*, Albert W. Schulthess^1^**

^1^ Department of Breeding Research, Leibniz Institute of Plant Genetics and Crop Plant Research (IPK), Corrensstr. 3, 06466, Gatersleben, Germany

^2^ Department of Genebank, Leibniz Institute of Plant Genetics and Crop Plant Research (IPK), Corrensstr. 3, 06466, Gatersleben, Germany

^3^ Institute for Biosafety in Plant Biotechnology, Julius Kühn-Institut (JKI) – Federal Research Centre for Cultivated Plants, 06484 Quedlinburg, Germany

^4^ Global Crop Diversity Trust, Platz der Vereinten Nationen 7, 53113 Bonn, Germany

*Corresponding author (reif@ipk-gatersleben.de).

1 Supplemental Tables

Table S1 Plausibility check of raw data based on species, annuality, availability, sowing time and physiology criteria.

| **Plausibility level** | **Criteria** | **Definition** | **Explanation** |
| --- | --- | --- | --- |
| Taxonomy† | Species | *Triticum aestivum* | Under the genus *Triticum*, IPK’s genebank is hosting 26 species and subspecies as well as species hybrids and botanically uncategorized accessions of diploid, tetraploid and hexaploid wheat.  Exclusively the free-threshing hexaploid wheat species *Triticum aestivum* was considered. Please note that some accessions are maintained as population. Only if all components of the population were determined as *Triticum aestivum* the accession was included into analysis. |
| Accession | Annuality | Winter type,  Facultative type | Exclusively accessions, which require vernalisation stimuli for flowering in combination with appropriate frost tolerance, were considered. |
|  | Availability | Available,  Currently not available,  Restricted available | Exclusively accessions, where genebank is able to hand over samples to a third person due to seed availability and legality were considered. Accessions which became extinct were omitted. |
| Record | Sowing time | Autumn sowing | Exclusively records with autumn sowing dates from September to the end of November were considered. Records of spring sown winter types were fully omitted. |
|  | Physiology | Trait in physiologically possible range | Flowering occurred during the 125th and 208th day of the year.  Plant height between 29 cm and 195 cm  Thousand grain weight between 12 g and 75 g |

†Traditional taxonomic classification (Dorofeev et al. 1979)

Table S2 The phenotypic diversity of IPK’s winter wheat collection in contrast to other collections sampled from Germany, Europe and world-wide origin. The investigated traits were flowering time in days after the 1^st^ of January (FT), plant height in cm (PH), and thousand grain weight in g (TGW), where Genotypes indicates the amount of genotypes in each study, Min is the minimum value, Max the maximum value, Mean the average value, Range the difference between maximum and minimum value and the $\boldsymbol{\sigma}_{\mathbf{G}}^{\mathbf{2}}$ genetic variance.

| **Source** | **German elite** | | |  | **Europe-wide collections** | | | | | |  | **World-wide collections** | | | | | | |
| --- | --- | --- | --- | --- | --- | --- | --- | --- | --- | --- | --- | --- | --- | --- | --- | --- | --- | --- |
|  | **Validation trials** | | |  | **Zanke et al. (2014a)** | | |  | **Langer et al. (2014)** | |  | **Neumann et al.(2010)** | | |  | **IPK winter wheat collection** | | |
|  |  |  |  |  | **Zanke et al. (2014b)** | | |  | **Würschum et al. (2015)** | |  |  |  |  |  |  |  |  |
|  |  |  |  |  | **Zanke et al. (2015)** | | |  |  | |  |  |  |  |  |  |  |  |
|  | **FT** | | **PH** |  | **FT**†† | **PH** | **TGW** |  | **FT**†† | **PH** |  | **FT** | **PH** | **TGW** |  | **FT** | **PH** | **TGW** |
| Genotypes | | 47 | 47 |  | 372 | 372 | 372 |  | 410 | 410 |  | 96 | 96 | 96 |  | 6,206 | 6,164 | 5,841 |
| Min | 153.29 | | 74.12 |  | 142.5 | 69.5 | 35.9 |  | 140.6 | 57.9 |  | 128.7 | 23.2 | 23.2 |  | 144.6 | 45.27 | 24.31 |
| Max | 163.86 | | 97.53 |  | 159.6 | 110.7 | 58.2 |  | 167.7 | 121.6 |  | 150.2 | 122.8 | 54 |  | 179.1 | 164.5 | 65.7 |
| Mean | 158.02 | | 84.98 |  | 151.4 | 87.2 | 45.4 |  | 157.6 | 76.1 |  | 138.7 | 80.4 | 36.1 |  | 160.6 | 113 | 46.86 |
| Range | 10.57 | | 23.41 |  | 17.1 | 41.2 | 22.3 |  | 27.1 | 63.7 |  | 21.5 | 99.6 | 30.8 |  | 34.5 | 119.23 | 41.39 |
| $\sigma_{G}^{2}$ | 1.98 | | 19.49 |  | 9.84† | 79.77† | 11.71† |  | 13.79 | 117.6 |  | 27.6 | 386.1 | 39.6 |  | 15.62 | 346.72 | 29.67 |

†reanalysis of the data; †† highly correlated heading date

2 Supplementary Figures


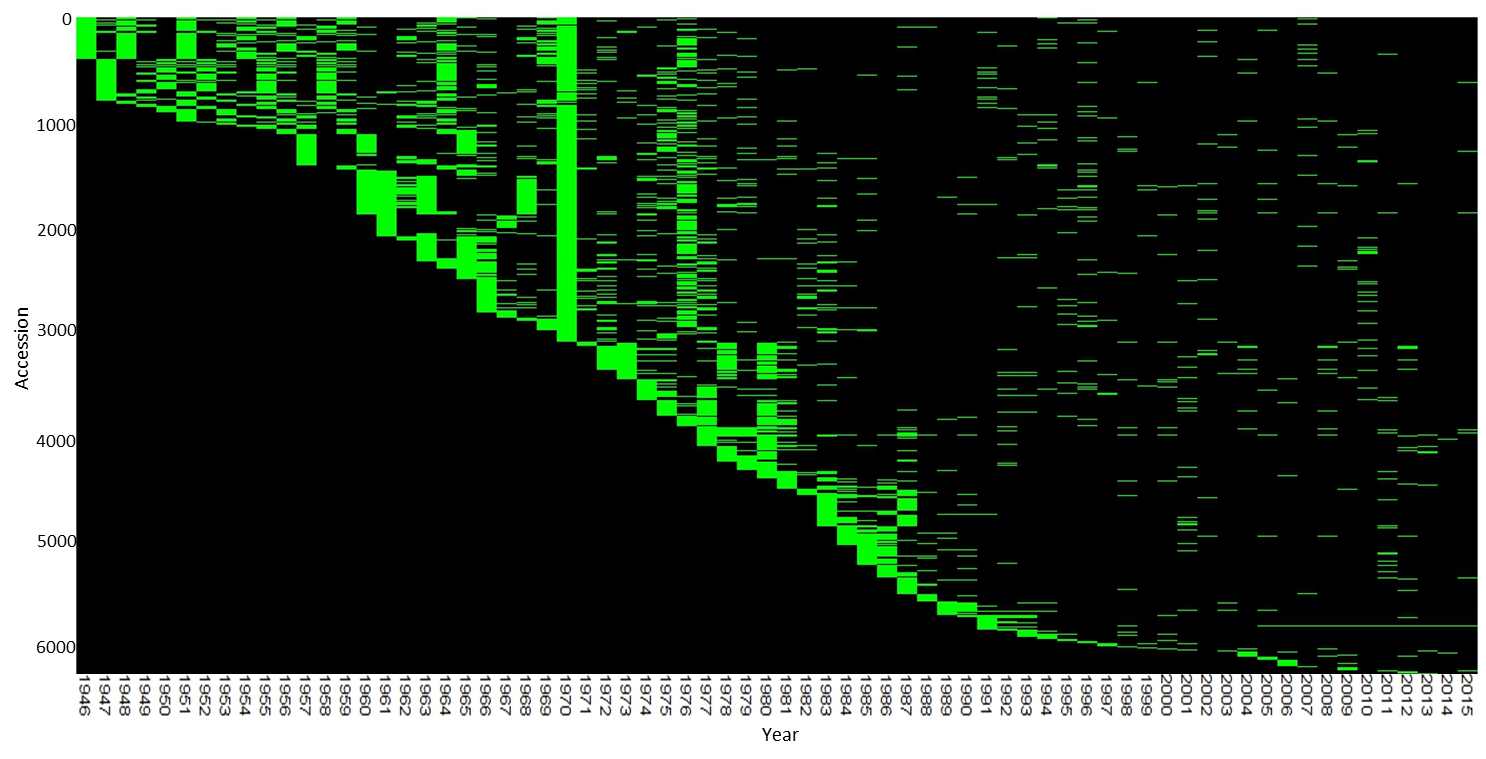
Figure S1 Data structure for 6,207 winter wheat accessions. In total 31,817, 31,139, and 25,808 data points were available for FT, PH, and TGW, respectively, gathered during seven decades of genebank regeneration between 1946 and 2015. Green bars indicate that an accession was regenerated in the respective year. Please note that due to limits in resolution green bars reflect several aggregated data points.


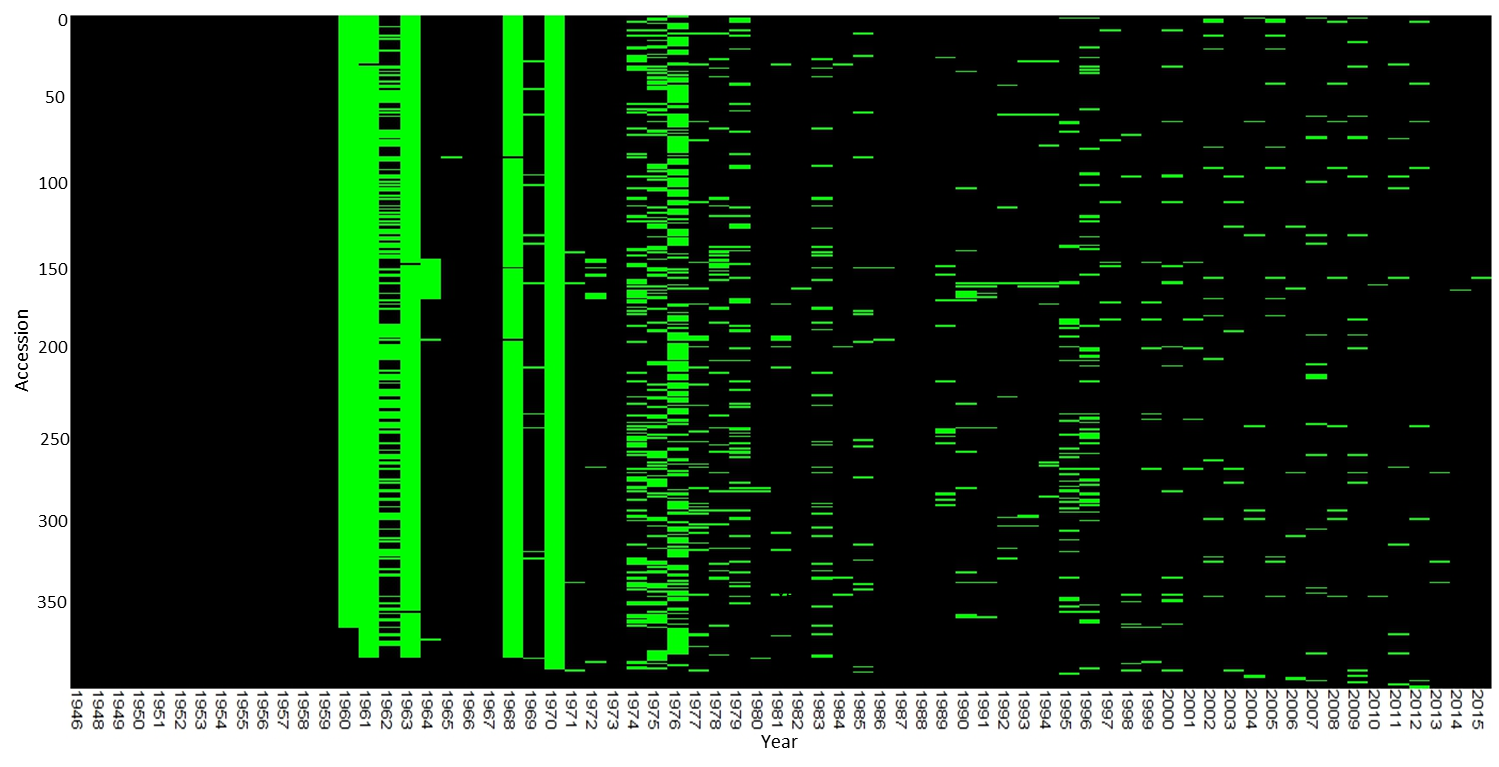
Figure S2 Collection pattern of 391 accessions originating from Iran collected in the time period ranging from 1946 to 2015. Green bars indicate that an accession was regenerated in the respective year.


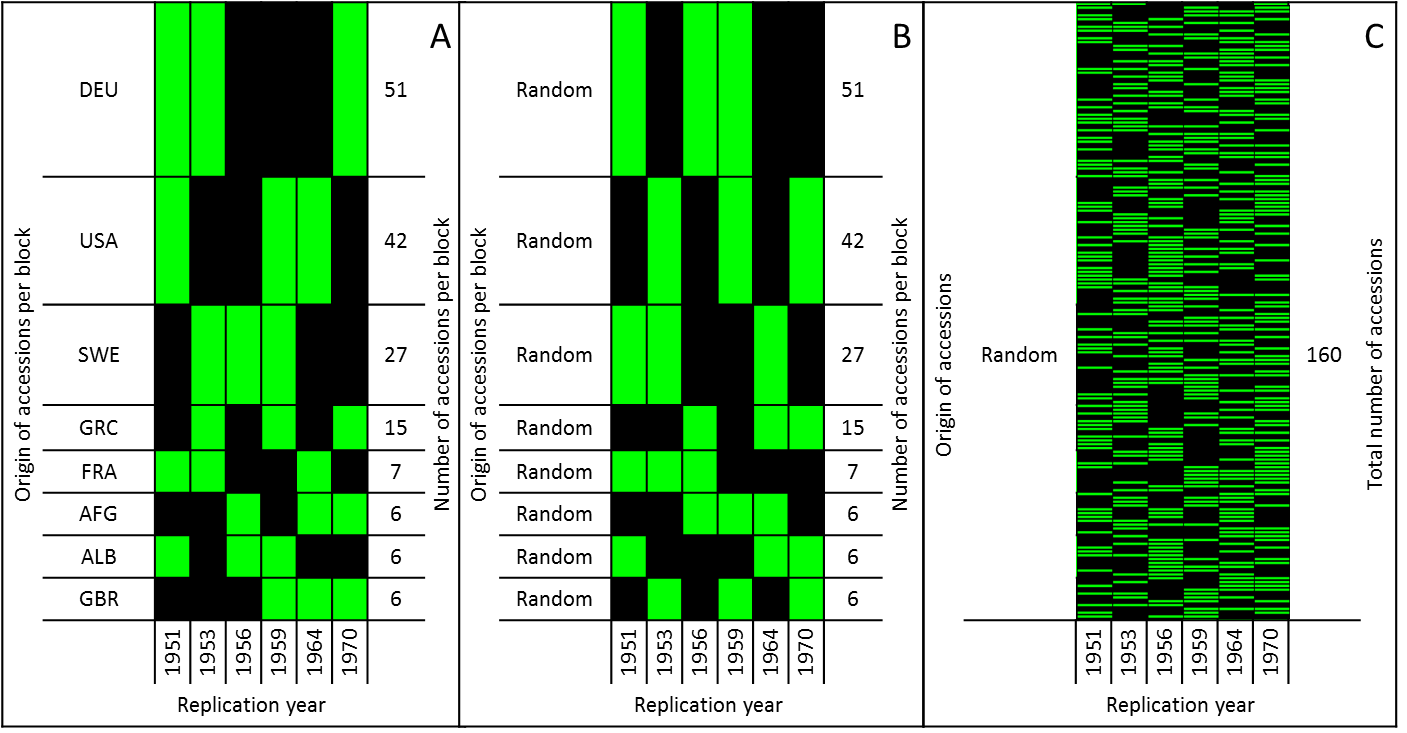


Figure S3 Illustration of the resampling Scenarios A, B and C. Based on an orthogonal historic sub-data set of 160 accessions regenerated together in six years, three out of six phenotypic records were sampled for each accession forming (A) blocks of accessions according to their geographic origin, (B) random blocks of accessions and (C) a block independent design. Green bars indicate presence of accessions, resampling was repeated 100 times.


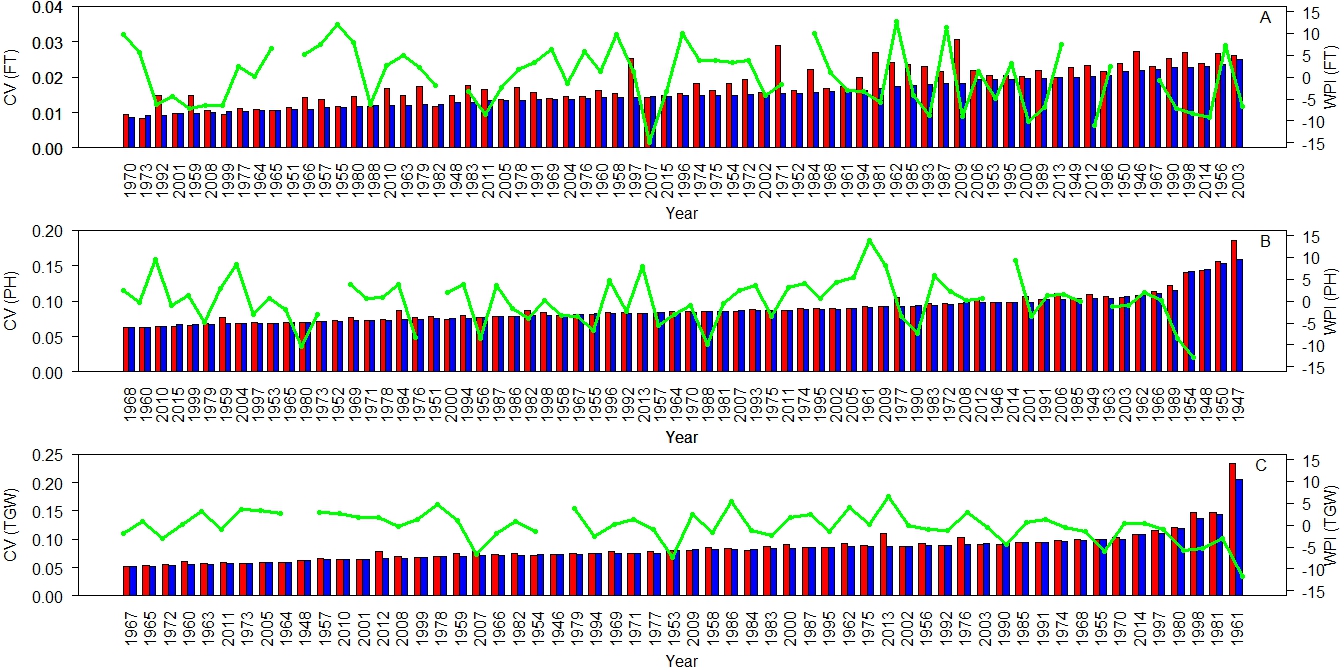


Figure S4 Coefficients of variation (CV) for residuals estimated before (red bars) and after (blue bars) outlier correction for (A) flowering time (FT); (B) plant height (PH) and (C) thousand grain weight (TGW). Weather parameter index (WPI) is shown as green line. Please note that the x-axis was ordered according to the CV after outlier correction.


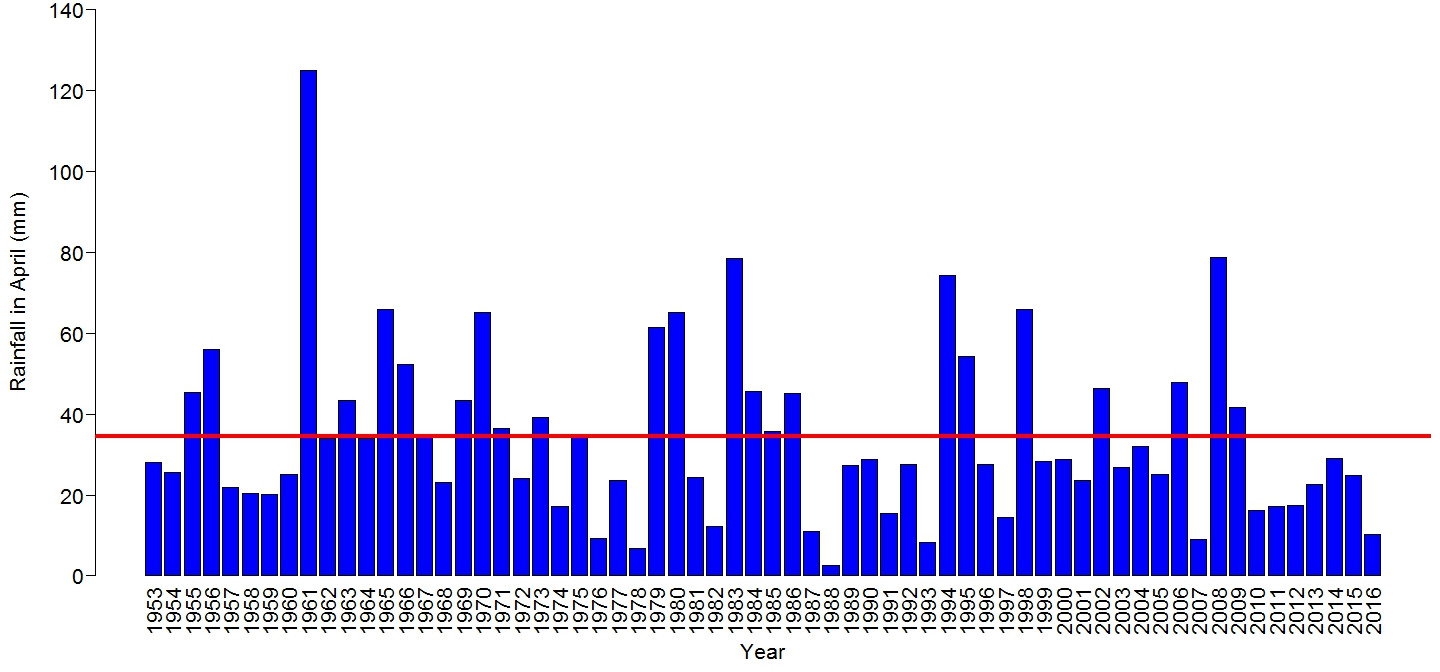
**Figure S5** Rainfall (mm) in April at Gatersleben between 1953 and 2016, red line refers to the local long-term average rainfall in April.


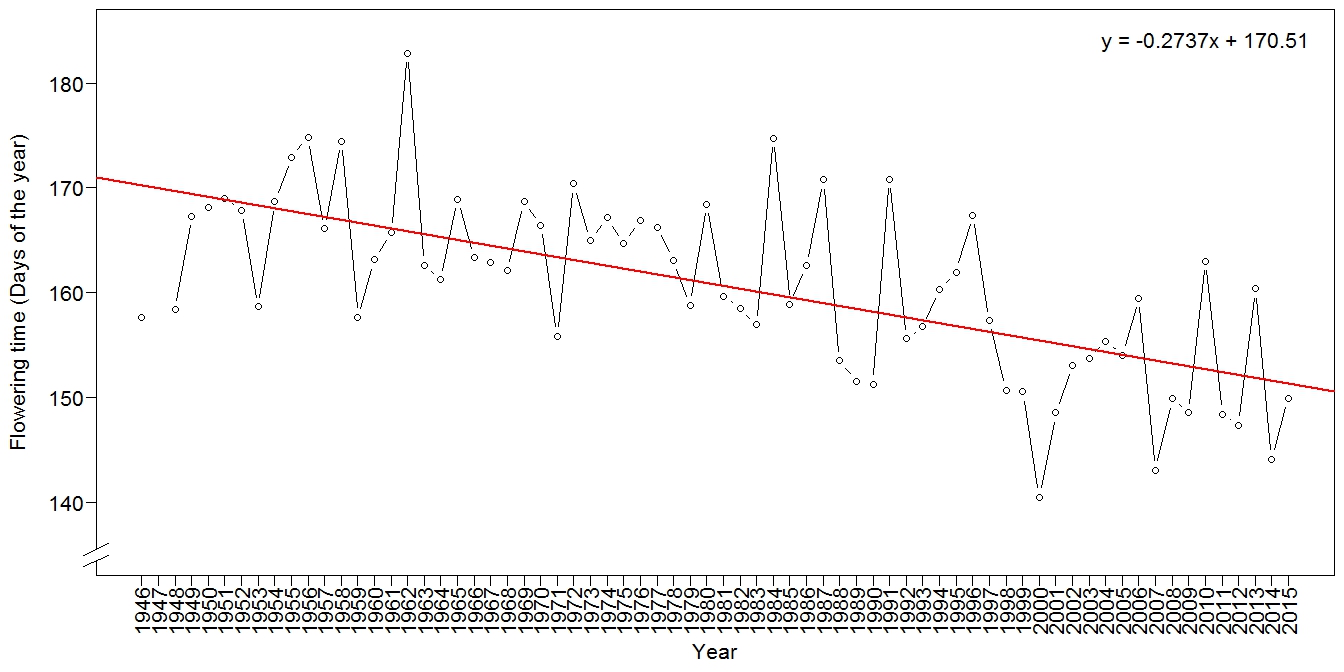
Figure S6 Temporal trend of flowering time between the years 1946 and 2015. Linear regression was performed between years and year effects for flowering time.

**3 Supplementary References**

Dorofeev, V.F., Filatenko, A.A., Migushova, E.F., Udaczin, R.A. and Jakubziner, M.M. (1979).” Wheat”. In: Dorofeev, V.F. and Korovina, O.N. (eds). Flora of Cultivated Plants, vol. 1. Leningrad, Russia.

Langer, S.M., Longinand, C.F.H., and Wurschum, T. (2014). Flowering time control in European winter wheat. Frontiers in Plant Science 5, 11.

Neumann, K., Kobiljski, B., Dencic, S., Varshney, R.K., and Börner, A. (2011). Genome-wide association mapping: a case study in bread wheat (Triticum aestivum L.). Molecular Breeding 27, 37-58.

Würschum, T., Langer, S.M., and Longin, C.F.H. (2015). Genetic control of plant height in European winter wheat cultivars. Theoretical and Applied Genetics 128, 865-874.

Zanke, C., Ling, J., Plieske, J., Kollers, S., Ebmeyer, E., Korzun, V., Argillier, O., Stiewe, G., Hinze, M., Beier, S., Ganal, M.W., and Röder, M.S. (2014a). Genetic architecture of main effect QTL for heading date in European winter wheat. Frontiers in Plant Science 5.

Zanke, C.D., Ling, J., Plieske, J., Kollers, S., Ebmeyer, E., Korzun, V., Argillier, O., Stiewe, G., Hinze, M., Neumann, F., Eichhorn, A., Polley, A., Jaenecke, C., Ganal, M.W., and Röder, M.S. (2015). Analysis of main effect QTL for thousand grain weight in European winter wheat (Triticum aestivum L.) by genome-wide association mapping. Frontiers in Plant Science 6, 14.

Zanke, C.D., Ling, J., Plieske, J., Kollers, S., Ebmeyer, E., Korzun, V., Argillier, O., Stiewe, G., Hinze, M., Neumann, K., Ganal, M.W., and Röder, M.S. (2014b). Whole Genome Association Mapping of Plant Height in Winter Wheat (Triticum aestivum L.). Plos One 9.
